# In Pursuit of the Gene: An Interview with James Schwartz

**DOI:** 10.1371/journal.pgen.1004308

**Published:** 2014-04-17

**Authors:** Jane Gitschier

**Affiliations:** Departments of Medicine and Pediatrics and Institute for Human Genetics, University of California San Francisco, San Francisco, California, United States of America

A few years ago, a friend suggested I take a look at a new book on the history of genetics written by independent scholar James Schwartz. Since my appetite for this kind of thing appears insatiable, I ordered the book and devoured it over a weekend. In *In Pursuit of the Gene*, Schwartz examines the brilliance of Mendel and Darwin; eavesdrops on the debates of Galton, Bateson, Pearson, Wendel, and De Vries; alights in the fly room of the early Drosophilists Morgan, Sturtevant, and Bridges; and settles at last on the highly unsettled Hermann Muller. This book is a must-read for anyone who is curious about genetics, and that means you, dear reader!

I soon contacted Schwartz (Image 1) for advice on a book project of my own, and our Skype conversation became so animated that I suggested we capture our dialogue with a formal *PLOS Genetics* interview. This came to pass when Schwartz visited San Francisco last May with his wife, Ann Hochschild (a faculty member of Harvard Medical School), and they kindly agreed to spend the night at my home to squeeze in a face-to-face interview.

**Figure:**
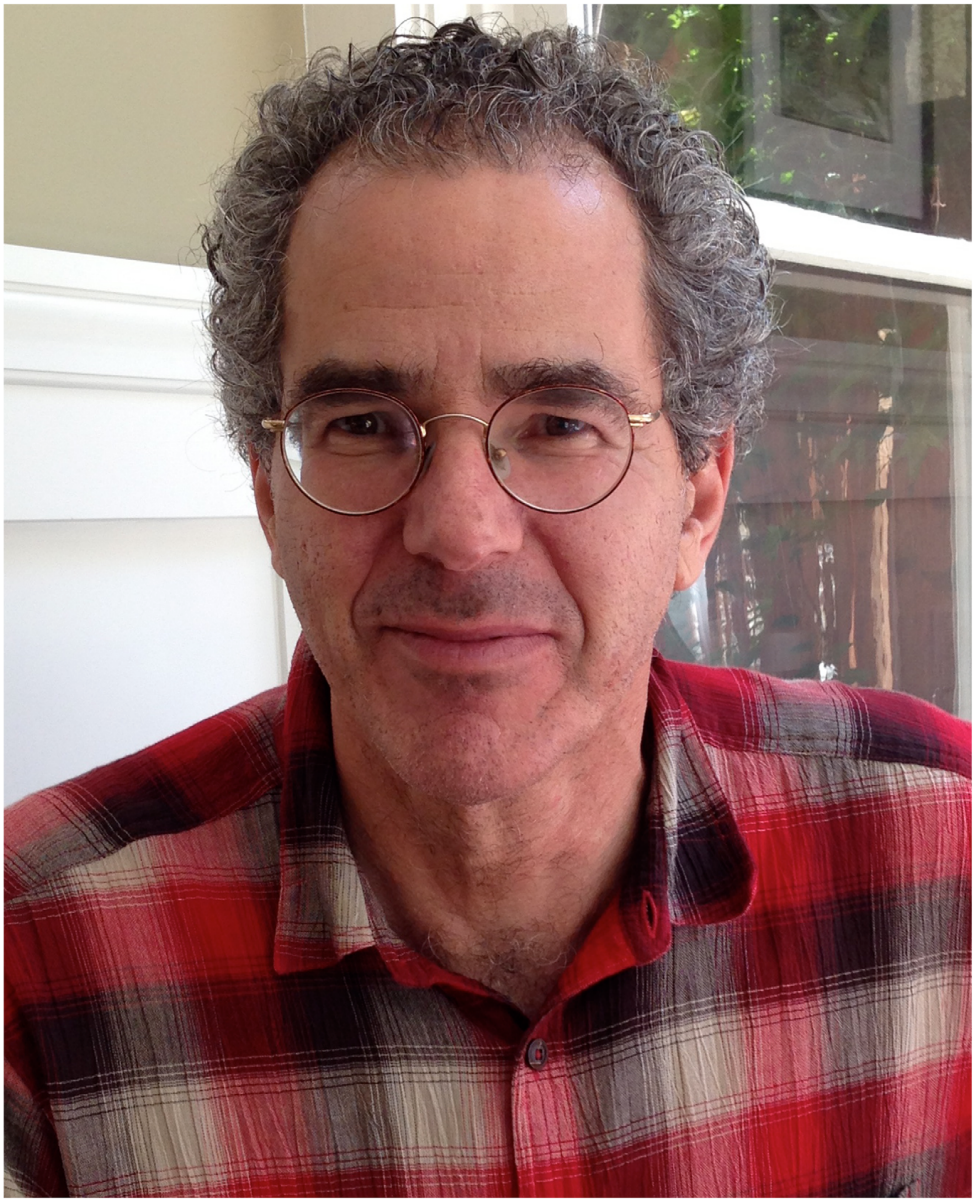
James Schwartz. Photo courtesy of Jane Gitschier.

I discovered that *In Pursuit of the Gene* was the unintended outgrowth of three articles Schwartz had written on the overarching question of the influence of genes on behavior, which included the sociobiology wars and the mathematical theories of altruism. In the early 2000s, Schwartz's plan to write a fourth article (on the then-contemporary science of association studies as applied to behavioral genetics) became derailed by a wee-hour discussion with the late David Cox, who pointed out that Hermann Muller had conceived of complex genetic traits decades earlier during his groundbreaking work on *Drosophila*. Schwartz became so captivated with Muller's story that he abandoned his article and turned his attention to the early history of genetics.

Schwartz may be uniquely suited to illuminate this history. Not only is he a very gifted writer, but he also trained in mathematics, as well as biology. He is also deeply curious about the psychology of the characters of this drama and how their particular personalities and circumstances provided grist for experiments and interpretations. I started out by asking Schwartz how he came to be a writer.

**Schwartz:** As an undergraduate in college I was an English major, but I also did pre-med [pre-medical studies] on the side. I wanted to be a psychiatrist. I was very ambivalent about the pre-med because it was so competitive and so unpleasant, particularly at Harvard, nearly entirely about how well you did and hardly at all about the beauty of the science.

In my senior year I took David Dressler's course in molecular biology, which was very historically based. He knew all of the characters, so it really came alive. To see how the results were found makes it so much more interesting and comprehensible. I became a teaching assistant the next year for that course, and I persuaded Ann to take it; she was a comp lit [comparative literature] major and had never taken any science in college. I knew she would love it.

**Gitschier:** So Dressler changed two lives!

**Schwartz:** Absolutely! He changed *many* lives.

By then I had entered graduate school [in molecular biology at Harvard], but I'm a purist, and I started working my way back through the sciences. I took physical chemistry first, then mathematical physics, and then straight math, which I found most compelling of all.

I ended up taking the entire math sequence—linear algebra, algebra, analysis, complex analysis; the complete undergraduate curriculum—and the biology faculty were starting to get mad at me. So I applied to MIT as a graduate student in math, and I was admitted.

**Gitschier:** How did that work out for you?

**Schwartz:** It was the sort of thing I had to do to work out a lot of things, and it was extremely valuable training in thinking analytically, but in the end, the whole experience of being a math graduate student was painful for me. Still, I saw it through. I think MIT is an amazing place, but I got overwhelmed by all the emphasis on greatness. In that department, it's all about how smart you are, and both the graduate students and the faculty talked about that all the time!

**Gitschier:** And after all of that, you leave math behind and start to write.

**Schwartz:** I had been writing literary stuff, short stories and a humor piece for *Poets & Writers*. Then I got very frustrated with a novel I had written. No one would publish it. It got so close! It was agony for me.

But my entire life, people said, “You should write about science. You have the math background; you have the biology background.” So I said, “All right, all right!”

*Boston Magazine* wanted a profile of Stephen Jay Gould and was offering a lot of money. I plunged into the subject and this whole fascinating debate over sociobiology and the wars that had taken place between Gould and Richard Lewontin on the one hand and E. O. Wilson on the other.

But I went *way* too far! I spent a lot of time with Lewontin and Gould, including taking their famous course on evolution, and also Steven Pinker. I also became good friends with Naomi Pierce, curator of butterflies and beetles at Harvard. She was very close to E. O. Wilson, and she was married to Andrew Berry, who was very close to Richard Lewontin. This was very exciting because I began to see that this was a story that could be told and had never really been told. They kept telling me various inside stories. By then [the article] had long gotten out of the domain of *Boston Magazine*, and *Lingua Franca* bought it and even provided an expense account!

I got in between Richard Lewontin and E. O. Wilson. For example, Wilson called me one night and said, “I have to read you this letter from Dick.” Wilson had given Lewontin his autobiography and Lewontin wrote back that he didn't like autobiography and wasn't tempted to read Wilson's. He wrote that we all create elaborate fictions about ourselves, but they were better left as waking dreams. This was extremely insightful, I think, and quite brilliant but also totally graceless. These two were at each other all the time. It was a very stressful period for me. These guys are very intellectually powerful and they hate each other! And I was caught right in the middle.

**Gitschier:** What about E. O. Wilson?

**Schwartz:** E. O. Wilson is great when he writes about ants, but he was totally guilty of bad science when he started extrapolating from ants to humans. The last chapter of *Sociobiology* is all about man. It is very provocative, and it deserved to be attacked, in my view, and they [Gould and Lewontin] took it on.

**Gitschier:** This piece, “Oh my Darwin,” is the first of a three-part series.

**Schwartz:** Yes. The second is called “Death of an Altruist” [later chosen for The Best American Science Writing 2001], when George Price met [William] Hamilton in England as Hamilton was formulating his theory on altruism. George was brilliant and mathematically very scintillating. He came up with a way of re-conceptualizing Hamilton's theory. Price's covariance equation is a beautiful equation; it relates selection at all levels—gene level, individual level, group level—with various factors, and it makes it plausible that there is group selection. Hamilton's papers are turgid, and the math is terrible! George brought a mathematical clarity to this mess.

[John] Maynard Smith, who is generally regarded as the inventor of evolutionary game theory, stole all of it from George Price, who had outlined the theory in a manuscript submitted to *Nature* that Maynard Smith had been sent for review. Hamilton hated Maynard Smith—a life-long hatred, because Hamilton believed that Smith had stolen from him as well.

But then I went to see Maynard Smith, and for reasons I'll never understand, he said, “I want to set the record straight.” He was very old, and he confessed! “Yes, it's true I read his proposal. I didn't exactly steal it, but when these ideas are there…I tried to get him to publish it with me.” And the first paper on evolutionary game theory is actually a joint paper between Price and Smith.

**Gitschier:** Oh dear. What became of Price?

**Schwartz:** He committed suicide in 1975.

**Gitschier:** Oh. What is the third article called?

**Schwartz:** “Population Genetics and Sociobiology.” The whole *Lingua Franca* plan was to have four articles, with the fourth one on genes and behavior and association studies. But then *Lingua Franca* went out of business, and by the time I got to four, I was sick of dealing with all the strife and anxiety that is part of writing about contemporary people who are still actively defending their turf. I had jumped into some pretty controversial people. I thought, “I can't take on another group of huge people!”

**Gitschier:** The egos!

**Schwartz:** The egos! My god! So out of control!

**Gitschier:** And so somehow you started in on *this* book.

**Schwartz:** Hamilton is called “the next Darwin,” at least among his friends, so I wanted to see what Darwin thought about all this [genetics and behavior], and I began to study Darwin and quickly got sucked into it. It has such history to it—a long and rich history.

**Gitschier:** But in researching that history, you can't actually meet the people.

**Schwartz:** They're dead, which is a relief! And they were great correspondents. Darwin had such wonderful letters.

Genetics is sort of a black box for Darwin. You don't need to know the actual mechanism to know his theory of evolution by natural selection. But it turned out that he was the inventor of the first modern theory of genes.

**Gitschier:** With his proposal of pangenesis and the gemmules.

**Schwartz:** Yes. “Gemmules” were the genes.

**Gitschier:** Before you approached *In Pursuit of the Gene*, did you already have in your mind the arc of this story, all the way to Muller? I'm just wondering if, like Athena, this book idea came out in one thought, or was it more iterative?

**Schwartz:** It was iterative, definitely. I was at one of those human genetics meetings around 2001, and I wanted to talk to David Cox because he was very early into the association studies. This was still when the fourth article was going to be about contemporary people and about genes and behavior.

I was last on his list for the day, and he stays up really late. He was drinking and drinking, but he was still very lucid. He said, “These guys like Eric Lander are saying this and that about complex traits, but it has a much longer history. Read Elof Carlson's book [*Genes, Radiation, and Society: The Life and Work of H. J. Muller*] and then you'll see that Muller had thought of this a long time ago.” So I got the book right away and read it.

Muller had seen the significance of complex traits and been the first one to show how you could map a complex trait, *truncate*, a phenotype in flies. David was into that, and then he brought up Darwin's cousin, Galton, and his studies of stature.

And he was vague about the connection between these various things, but if there was an Athena moment, it was that night with Cox, because I was already aware of Darwin's complicated relationship with his cousin and fascinated by Galton for a long time. He reminded me of myself in many ways. He was very concerned about being smart, which resonated with me, and he also had a similar kind of math fixation.

Even before I met David, I had been reading Pearson's biography of Galton, which no one reads: *The Life, Letters, and Labours of Francis Galton*, in three volumes. Galton's mother, Violetta, wanted him to be a prodigy. She was the daughter of Erasmus Darwin, who was a brilliant philosopher, poet, and physician, a member of the Lunar Society.

Galton desperately wanted to go to Cambridge and study math. And his father said, “Just become a doctor!” Actually that is similar too! [We laugh, because Schwartz's father is a physician.]

He did prevail, with Darwin's help, and his father let him study math, which he was really ill equipped to do. By the spring of his second year Galton found that he couldn't keep up with it.

**Gitschier:** Why did he do math then?

**Schwartz:** He had this need to prove he was brilliant.

**Gitschier:** And math is a metric for that.

**Schwartz:** Right, and he was intensely interested in the metrics for that. Even in college, Galton had a nervous breakdown over this “am I smart enough?” issue. Then the identical issue was rekindled twenty years later, when he wrote *Hereditary Genius* in his late 40s. Again he had a nervous breakdown and the symptoms of the second breakdown were almost identical to the first!

When he first read Darwin's work on pangenesis, Galton thought, “This is a brilliant theory—a particulate theory of inheritance.” And he totally got that you could make a polygenic model in which intelligence would be normally distributed. He immediately saw that this would be enormously helpful to him. That it is a mathematical way to conceptualize his prejudices.

**Gitschier:** Even though Darwin himself didn't mathematically conceptualize this.

**Schwartz:** No, Darwin wasn't good at math.

**Gitschier:** But then Galton also sets out to test Darwin's hypothesis of gemmules with his rabbit blood transfusion experiment.

**Schwartz:** He doesn't do that dispassionately.

Galton loved Darwin, and he admired him to death. There was also a whole social side to this, because the Darwins were *the Darwins* and a whole network of cousins and relatives. He wanted to be part of that group more than anything, a real social climber.

But he was upset. He saw that Darwin's pangenesis theory allowed for the environment to act on the genes themselves. The gemmules were circulating around, and if you became super strong, these little gemmules were sent back from the muscles to the reproductive organs [to pass on that strength to the next generation]. When I started to read Darwin, it took me a year to get my head around the degree to which Darwin would buy some obvious nonsense, like that scars were inherited.

**Gitschier:** The whole Lamarckian…

**Schwartz:** …framework. He was a Lamarckian beyond belief.

**Gitschier:** I knew none of this about Darwin [before I read your book].

**Schwartz:** It's shocking to find out.

**Gitschier:** Actually, it's interesting how wrong Darwin was on so many points!

**Schwartz:** He was driven by his first and primary passion—evolution by natural selection—and when that theory was really saliently critiqued by one of his contemporaries, who showed that under the mixing theory of inheritance—whereby a tall man and a short woman give rise, on average, to medium-height children—a population would quickly run out of variation and, therefore, evolution by natural selection would have to grind to a halt, Darwin came up with pangenesis so that the environment could generate variation.

**Gitschier:** So he wrote another book [*The Variation of Animals and Plants Under Domestication*] in which he proposed pangenesis, in response to the criticism.

**Schwartz:** Absolutely. Directly in response.

That bothered Galton a great deal; he wanted intelligence to be hereditary. Why? He wanted—like many of these guys—to be special, and it to be a God-given thing that he was special. He didn't want it to depend on the fact that he had rich parents and every opportunity on earth. Rather, that the rich were rich because they had better genes and therefore prospered. It's a repellent theory.

I'm not sure it is right to trace this all the way up to contemporary sociobiology, but there is that same feeling. It's what Pinker and Wilson were saying and what got Larry Summers in trouble: that women aren't as good in science genetically.

**Gitschier:** Right. So the rabbit transfusion experiment fails, and then Darwin says, “Well, I didn't say that it had to be in the blood!”

**Schwartz:** Darwin's looking a little bit weak at that point!

**Gitschier:** And once it didn't work…

**Schwartz:** Galton was off and running.

**Gitschier:** Then, he's thinking, “This supports eugenics.”

**Schwartz:** He's already a eugenicist. By 1865 he had written an incredibly eugenic article for Macmillan's.

There is a deep point here, I think, about art and science and how they work: Galton was obsessed with his intelligence. He had to prove that he had hereditary intelligence, that he was very special, and this kept on causing him huge problems in his life. Yet, he was dogged and incredibly energetic and imaginative in trying to prove all of this. So he was actually working through this deep psychological issue he had and that led to this creation. Because, whether you like what Galton stood for or not, he is the inventor of the concept of correlation, and regression, and much else.

My point is that this is what motivated him, and somehow what motivates all artists is some unresolved childhood thing that they keep going back to over and over.

**Gitschier:** Are you talking about artists or scientists?

**Schwartz:** I think they are the same in this way. Deep, creative productions have to tie into deep, unconscious conflicts. Now, I'm surprised I'm bringing this up, but I think this was in the back of my head when I was writing about Galton. It's not really “my” theory, but it comes from Freud's book on Leonardo, and the idea was later elaborated in the writing of Hanna Segal.

It's almost an irony that what was hanging him up was that he didn't have this very special kind of “wrangler”-like math gift [the nickname for the top mathematics students at Cambridge], but meanwhile, he was building his quincunx and he was doing all of these incredible things! He kept on working and working and producing this remarkable creative output, which turns out to solve the problem for him. I do think that at the end of his life he was a lot better.

He was a very old man when he was mediating between [William] Bateson and [W. F. R.] Weldon on the evolution committee. Bateson's discontinuous evolution was how he [Galton] wanted it to be. It was feeding into his own issues. He believed that regression to the mean would destroy all the genetically super-endowed people. So he needed to have these sudden leaps, the “sports” [as mutagenic events were known at the time].

**Gitschier:** Now, talking about sports and the monstrosities, let's bring in [Hugo] De Vries.

**Schwartz:** That's why Bateson was so attracted to De Vries. Bateson was obsessed with discontinuous evolution. He met De Vries, whose whole theory was that a single pangene could change or even create a new species. What could be more discontinuous?

But then Bateson discovered Mendel, and said, “Oh my god, this is the discovery of the century!” Whereas De Vries, because he was immensely vain, just wouldn't let go of *his* mutation theory, which was wrong, even to the very end of his life.

**Gitschier:** De Vries was right in the sense that there are mutations and discontinuity, but what was happening in the organism he was actually studying [*Oenothera*] wasn't what he was espousing.

**Schwartz:** It was really Mendelism, which is what Muller shows. It was the accumulation of a number of recessive mutations on these balanced lethal chromosomes.

**Gitschier:** Which were revealed…

**Schwartz:** …in a snap as soon as they were set free.

**Gitschier:** I felt so often that the characters were entrenched in their thinking. It seems in hindsight to be such a small point…

**Schwartz:** You're saying, why couldn't he just move on? Because it's more than his work. These people are connecting to deep conflicts that they have. Now with De Vries, his conflict stemmed from the fact that his father was the head of the Supreme Court in the Netherlands, and the son wanted to make a great splash. By the early 1900s, De Vries was more important than Darwin! He did a US tour where he set every city on fire.

**Gitschier:** Let's talk a little bit about Bateson. He seems amazing!

**Schwartz:** Bateson was my favorite character in a way. His correspondence is all on microfilm [at the American Philosophical Society], and it's enormous. His observations about people are so astute.

**Gitschier:** He has one quote in here that “Morgan is a blockhead!”

**Schwartz:** He could not abide [Thomas Hunt] Morgan. He hated Morgan because Morgan is so unsettled and not at all sophisticated about people.

**Gitschier:** I also loved the story about how Bateson researched the coat colors of horses in Britain in his pursuit of evidence for Mendel.

**Schwartz:** That's a great story. And in terms of researching the book, that was also one of my favorites. Bateson meets [Charles] Hurst in the Café Austrian just before the big confrontation with Pearson over coat color. Hurst comes in and says, “Let's order a bottle of champagne.” Bateson, horrified, says, “What, we can't have champagne, we have to have clarity of mind!”

They get in the cab, and Bateson looks at the cab's horse, a bay—which is a chestnut with a brown tail—but the horse had an alarmingly dark coat. Bateson suddenly doubts the entire thing. He's going nuts. And Hurst says, “No, no, this is a special kind of chestnut, called a liver chestnut.” Every horse on the street becomes a test case! Discovering that whole story was a fantastic moment for me.

**Gitschier:** Where did you find it?

**Schwartz:** Also in the archives of the American Philosophical Society. It was in a book that Hurst's wife had written—more than a thousand typed pages on this terribly thin onion-skin paper. It was clear that no one had ever read it.

By no means was Bateson the most impressive scientist, but he was a free thinker and he had great honesty. He was pursuing these tracks; he kept going down dead ends and changing his direction. He saw how important Mendelism was; he made it his life's mission to make sure that everybody appreciated it. Because Mendel, of course, was the genius, the Mozart behind this all, because he just *saw* it, the way genetics worked.

**Gitschier:** I would have thought you were going to say that your favorite character in this drama was Muller.

**Schwartz:** From a literary point of view, the best writer and the person most full of insight is Bateson. Scientifically, the most exciting is Muller. His mind! The balanced lethal idea is so beautiful and so incredible, and these ideas would occur to him in a flash. In *Drosophila*, there are so many markers involved and so many things to keep in mind. He was just otherworldly in his ability to design experiments.

**Gitschier:** So, you read all these papers.

**Schwartz:** Oh, totally! I spent weeks with some of them. The 1927 paper that shows X-rays cause mutations is so beautiful. And then again with the complex trait—*truncate*. Muller's view of life was so complex.

**Gitschier:** But what about his nutty personal journey? He's frenetic. He feels excluded from the Morgan inner group, but then he goes to Rice [University] and he brings Edgar [Altenberg] out. I would think, “I've got my best friend here, I'm going to stay.”

**Schwartz:** But you're not as neurotic. He's hugely neurotic, ceaselessly driven by some internal, unresolved psychological issues. Muller, Galton, De Vries – all these guys are driven by some force that they don't even understand themselves.

From Rice, Muller goes back to Columbia for two years, hoping to be accepted and loved. But there was some problem, so then he went to the University of Texas in Austin, which was a very good job. You might have thought he could be happy there. I don't want to give credence to the Morgan-fly-group view of him that he is a troubled guy who caused trouble, because I don't think that is true. He had a lot of friends, was very generous with his ideas—but wherever he went…

**Gitschier:** Speaking of generous, when Muller is still a grad student, he forms a little reading group, that included Bridges and Sturtevant when they were undergraduates—and he wrote that little paper…

**Schwartz:** “Erroneous Assumptions Regarding Genes.” Unbelievable – he was 22! That's a beautiful paper.

**Gitschier:** Where did you find that?

**Schwartz:** That's in Muller's collection of papers. Never published, but he saved everything.

**Gitschier:** So with Muller zipping off to Berlin, then Leningrad, and then Moscow, I would have thought that these papers would have long-since disappeared.

**Schwartz:** All the personal papers from the Russia period were destroyed by his wife, to protect him. This was during the McCarthy era, when Muller had returned to the States and finally landed a job at Indiana University Bloomington.

It's a pity. His personal life was so troubled. This is what is bothering me now. For the next book—I do want to go on with this story—but how you can deal with Muller in Russia? There was so much going on in Russia and so much politics involved, and the intersection of politics and science is so stark and revealing.

Americans don't understand it. Stalin destroyed all the geneticists and destroyed the biological sciences there, an evil and horrible psycho-killer. On the other hand, a lot of what Stalin was saying was exactly right. He was being criticized for directing science and writes an editorial in *Izvestia*, the main daily paper: “The Americans claim that they have true intellectual freedom, but all their science is run by a small group of capitalists for the benefit of corporations.” And he's partly right!

I want to write the story about this Stalin period, and Muller's right at the center of it, but there are all of these personal, weird disharmonies.

**Gitschier:** I also found it fascinating to read about Muller going off to Germany and Russia and taking all his fly stocks!

**Schwartz:** I love the fly stock stories! Wherever he goes, the flies are like an appendage to him. And later, when he's taking the seaplane from Portugal to America and he accidentally leaves the flies, which are in a breadbox, on the tarmac—he thinks he has forgotten them—and he's begging the pilot to turn around!

**Gitschier:** Here we are escaping from World War II, and we're going back to get the flies!

**Schwartz:** I almost hear him saying, “You absolutely must turn back.” They did radio back and found out the flies weren't on the tarmac. They were brought to the galley because they thought it was actually bread!

**Gitschier:** How did you learn that?

**Schwartz:** That's through Thea, Muller's second wife, who wrote unpublished memories of Europe. They are somewhere buried in the Lilly archives [at Indiana University].

Then there is Muller's personal life through all of this—how do you reconcile …

**Gitschier:** Well, here's Muller's wife's lover coming with them to Leningrad, and he continues to work with this guy!

**Schwartz:** The thing about Muller is that when he's in Texas, he's writing a paper about eugenics, and his bottom-line idea is that women are like slaves. In this kind of capitalistic environment, we're never going to un-enslave women, the only way this can happen is in a communistic society.

**Gitschier:** He appreciated that, unlike Galton…

**Schwartz:** He's the opposite side!

**Gitschier:** …that among the poor there might well be some geniuses—he wanted everybody to be given the *same* opportunity!

**Schwartz:** Yeah! Exactly. His point was how to actually get at what the true genetics was telling you.

**Gitschier:** I get the feeling that you are a lot like me, in that when I'm researching or interviewing someone, I almost feel like I'm now a part of his or her life in some way.

**Schwartz:** Yeah. You identify with the subject.

**Gitschier:** By immersing myself in your book, I feel such a strong connection. You've gone through all this hard work to read the original papers and correspondence and to look between the lines, and now I get to read this and to have access to these people.

I'm working on this book on splicing, and I'm reading all the papers, and I feel that there are some holes in my understanding, but I kind of get it, and I talk to the people about it. I feel that this is such a privilege to be able to sit down and talk to these people, and they appreciate that I have taken the time to try to understand this, I think. It is an incredible opportunity! There is something so meaningful about this process.

**Schwartz:** Yes, it's very nurturing, almost spiritual. I agree with all of what you've said; in particular, when you go into these people's lives, you feel you know them very closely. You lose your objectivity because you're rooting for them. How could he say that? What did he really mean? But it's memorializing them, too. You want to capture these incredible moments they had. So it's a good thing to do, isn't it?

**Gitschier:** You are honoring them.

**Schwartz:** You are honoring the best of human nature.

